# Social inequalities in the use of physiotherapy in women diagnosed with breast cancer in Barcelona: DAMA cohort

**DOI:** 10.1007/s10549-023-07191-9

**Published:** 2023-12-28

**Authors:** Rocio Cogollos-de-la-Peña, Anaís Álvarez-Vargas, Fernando Domínguez-Navarro, Albert Espelt, Laura Fuentes-Aparicio, Rosa Puigpinós‑Riera

**Affiliations:** 1 Faculty of Health Science, Universidad Europea de Valencia, Valencia, Spain; 2 Departament d’Epidemiologia i Metodologia de Les Ciències Socials I de La Salut d’Umanresa, Universitat de Vic-Universitat Central de Catalunya, Manresa, Catalonia Spain; 3 Department of Physiotherapy, Faculty of Physiotherapy, University of Valencia, Gascó Oliag 5. 46010, Valencia, Spain; 4 Departament de Psicobiologia i Metodologia de Les Ciències de La Salut, Bellaterra, Catalonia Spain; 5 Centre for Biomedical Research in Epidemiology and Public Health (CIBERESP), Madrid, Spain; 6 Physiotherapy in Motion, Multispeciality Research Group (PTinMOTION), Department of Physiotherapy, University of Valencia, Valencia, Spain; 7 Agència de Salut Pública de Barcelona, Plaça Lesseps, Barcelona, Catalonia Spain; 8 Biomedical Research Institute Sant Pau (IIB Sant Pau), Barcelona, Catalonia Spain

**Keywords:** Breast cancer, Rehabilitation, Physiotherapy, Socio-economic profile, Epidemiology

## Abstract

**Purpose:**

This study aimed to analyze social inequalities in the use and access of physiotherapy service and its clinical and socio-economic determinants in women diagnosed with breast cancer in the hospital network of Barcelona.

**Methods:**

Data from 2235 women belonging to the mixed (prospective and retrospective) DAMA Cohort were analyzed, including demographic, socio-economic, clinical, and breast cancer treatment outcomes. To determine the influence of such variables on access to physiotherapy, different Poisson regression models with robust variance (obtaining Prevalence Ratios and confidence intervals) were estimated.

**Results:**

Although when experiencing different chronic and acute symptoms, only between 20 and 35% of women visited physiotherapist. Two out of 3 women reported to have received insufficient information about medical care and rehabilitation. Age of women, job occupation, education level, having a mutual or private insurance, as well as outcomes related to breast cancer, appear to be factors influencing the access to physiotherapy.

**Conclusions:**

Social and economic inequalities exist on the access to physiotherapy by women diagnosed with breast cancer, which is generally low, and may clearly impact on their functional recovery. Promoting strategies to reduce social bias, as well as improve communication and patient information regarding physiotherapy may be of interest for a better health care in breast cancer diagnosed women.

## Introduction

Breast cancer is the most prevalent type of cancer among Spanish women, with a high incidence in those of working age [1]. The advances experienced during last decades in both early detection and treatment of breast cancer have resulted in a satisfactory reduction in mortality, leading to a survival rate of around 90% [2]. The improvement in survival rates highlights the necessity of restoring an adequate level of functionality and quality of life that enable women to reintegrate into working activities. Nevertheless, a substantial number of breast cancer survivors experience physical and functional limitations due to the received treatments, the disease itself, the associated fatigue, and the decreased physical capacity; that may persist up to 6-years post-surgery [3]. Physical-functional impairments include musculoskeletal-pain, reduced shoulder mobility, diminished upper-limb strength, lymphedema, and chest wall tightness [4–6]*.* Overall, they have a negative impact on women’s psychological health, as well as placing a greater demand on health and social services [7].

Physiotherapy interventions, including exercise and manual therapy, have shown to be useful in increasing functionality and improving symptoms in breast cancer survivors [8, 9]. Furthermore, benefits in terms of muscle strength, joint mobility, and cardiorespiratory fitness were described [10].

However, despite the scientifically reported benefits, on the pragmatic level, access to and use of physiotherapy for women diagnosed with breast cancer is hampered by challenges associated to the organization of healthcare resources [11] and the lack of information provided by some medical professionals [12].

Overall, diverse studies indicate that only around 9–20% of patients who have undergone breast cancer-surgery have adequate access to physiotherapy and rehabilitation services [13, 14]. As a result, a high percentage of women are missing out on these potential functional benefits [15]. Likewise, current evidence suggest that access to health care systems may not be equal for all women with breast cancer, with socio-economic factors, such as occupation, and level of studies playing a relevant role [16, 17], especially during the period after active treatment [18] where physiotherapy interventions are applied.

Nevertheless, to the best of our knowledge, the access and use of physiotherapy services by breast cancer survivors treated in health centers in the city of Barcelona has not been collected in a global and detailed manner, considering the clinical and socio-economic influential factors. Based on this analysis, it would be possible to identify gaps and areas for improvement to improve the quality of rehabilitation for women diagnosed with breast cancer. Thus, the aim of this study was to determine the degree of access to physiotherapy services by women diagnosed with breast cancer in the hospital network of Barcelona, as well as to analyze which factors (demographic, socio-economic, and clinical-related) were associated.

## Materials and methods

### Design and setting

The present research is a mixed cohort study (prospective and retrospective) nested in the DAMA cohort (acronyms of women with breast cancer in Catalan) [17], using a convenience sample on a total of 9771 women diagnosed with breast cancer. The primary aim of this cohort is to establish a foundation for the scientific and medical examination of the sociodemographic, economic, clinical, and lifestyle characteristics of these women, as detailed in previously published articles from DAMA cohort [17, 19, 20].

Retrospective information was collected from the clinical histories from the moment of breast cancer diagnosis (from 1st January 2003) to the present. Prospective information comes from the questionnaires conducted during the study period (from January to November 2016). The study encompasses the 4 main hospitals in the urban network of Barcelona (Hospital del Mar, Hospital Clínic, Hospital de la Vall d’Hebrón y Hospital de Sant Pau) and was coordinated by Agència de Salut Pública de Barcelona (ASPB). The data available for this study comprised information related to access to physiotherapy and its clinical and socio-economic determinants from 2235 women. This research received the approval of the Ethics Committee granted by Hospital del Mar (Parc de Salut Mar 2015/6499/I).

### Participants

As described in previous articles [19], DAMA cohort is composed of women older than 18 years old diagnosed and/or treated with cancer at different stages, and with information from the time of diagnosis onwards (1st January 2003 and 31st December 2013). The participating women were users or patients of the aforementioned 4 public hospitals in Barcelona. Inclusion criteria for the mentioned cohort were as follows: (I) women over 18 years of age, (II) hospital admission with a primary diagnosis of breast cancer, and (III) diagnosed or treated at any time during the study period and who had signed the informed consent form. Likewise, we excluded those subjects who (I) died of any other cause before 2015, (II) were diagnosed with any other type of cancer before breast cancer diagnosis, and (III) lived outside Catalonia, due to difficulties in carrying out the follow-up.

### Procedure

The first contact with the potential participants (9771 women meeting inclusion criteria) was through the medical staff of the admitting hospitals, who sent a letter, via conventional mail, with detailed information regarding the nature and the objectives of the study. Two copies of the informed consent form were attached in a postage-paid envelope, so that those who voluntarily decided to participate could keep one copy and deliver the other signed to the ASPB in the stamped envelope. Thus, 2760 informed consents were received. The women from whom informed consent was received were telephoned by the ASPB member. In that call, the details of the study were explained again, and they were given a Welcome Questionnaire, from where sociodemographic data were obtained to determine the profile of women in the cohort.

Subsequently, a General Questionnaire was sent by conventional mail. Parallelly, they were given the opportunity to respond through a virtual platform. The estimated time to complete the questionnaire was 30–45 min. The General Health Questionnaire included 6 sections: (I) personal data, (II) socio-economic characteristics, (III) clinical symptomatology, (IV) data on breast cancer and its treatment, (V) lifestyles, and (VI) emotional well-being. If no response was obtained within a period of 1 month, participants were recontacted to complete the questionnaire, excluding from the study those who, after this reminder, did not make it.

### Information sources:

Data were obtained from (I) medical records, to retrieve information from the clinical history; (II Welcome Questionnaire) to determine the social profile; (III) General Questionnaire, providing information for clinical symptomatology and usage of physiotherapy.

### Outcomes

In accordance with the objective of the study and the available information, data regarding access to physiotherapy, socio-economic characteristics, acute and chronic symptomatology, as well as breast cancer status and its treatment were extracted and analyzed.

Socio-economic section includes questions about age, level of education attained (no education or primary, secondary, and higher education), and their own occupation and that of their partners or cohabitants (following the National Classification of Occupations: manual workers, intermediate occupations, directors and managers). In the case of different levels of occupation between the woman and the partner or cohabitant, the higher of the two levels was assigned. These data were used to determine social class [21]. In the section on clinical symptomatology, we asked about the presence of symptoms of cervical pain, chronic low back pain, chronic tendinopathy, arm pain, lymphedema, loss of arm strength, and loss of grip strength in the hand. The data section on breast cancer and its treatment included cancer stage (in situ, early-stage tumors, local tumors, metastatic tumors), years since diagnosis (0–3, 4–6, 7–9, 10–26) and number of relapses (none, one or more than one). From the emotional well-being section, it was only included the Question 48 (*In the last 12 months, have you visited any of the following health or social-health professionals?*”) which was considered as dependent variable of the study. The complete questionnaire could be consulted in the following link: https://www.aspb.cat/wpcontent/uploads/2016/11/Questionari-Cohort-DAMA.pdf

### Statistical analysis

The statistical analysis was carried out with the Stata program. Firstly, a descriptive table was made with the data from the 6 sections of the survey, segmented according to the dependent variable: whether they had visited a physiotherapist in the last 12 months. A test was used to calculate whether there were significant differences between the two groups for each of the independent variables from the sections: socio-economic characteristics, general health and medical history, and data on breast cancer and its treatment. Next, a specific descriptive study was carried out for those who did attend physical therapy services.

The proportions of each independent variable were also calculated for those who had attended the physiotherapist, and finally, a multivariate Poisson regression model with robust variance was performed to find the variables associated with going to the physiotherapist [22] The significance level selected was *p* value < 0.05.

## Results

### Flow chart of participants

**Fig. 1:**
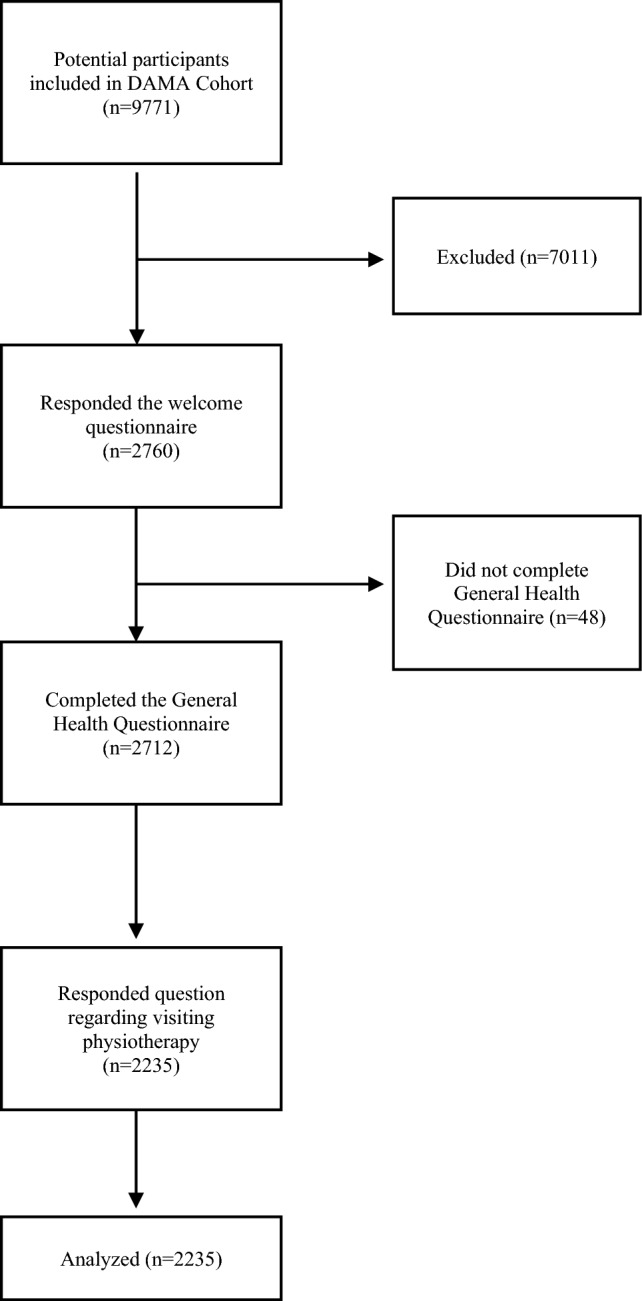
Flow chart diagram of the participant in the study

From the total DAMA cohort population, 2760 subjects responded the telephone survey (welcome questionnaire) and were sent the General Health Questionnaire. Of these, 98.26% (2712 women, mean age 62.2 years) completed the questionnaire and were included in the study. For the present study, it was analyzed the data from 2235 women who answer the Question 48 about access to physiotherapy. Flow chart diagram of participants is presented in Fig. 1.

### Access to physiotherapist, socio-economic, and breast cancer outcomes

Question 48 of the Emotional well-being section, which referred to access to physiotherapy: “In the last 12 months, have you visited any of the following health or social-health professionals?” was answered by 2235 participants, of whom, only 409 (18%) checked the “yes, by a physiotherapist” option.

Table 1 shows the descriptive values for sociodemographic and breast cancer outcomes for women visiting physiotherapists. Women under 62 years (64,3%), with superior level of education (53.8%), with directors and managers-related occupations jobs (40.7%), and having mutual or private insurance (64%) were those who most frequently visited physiotherapist. Likewise, those with local tumor (54,1%), and with more than one relapse

**Table 1:** Descriptive values for sociodemographic and breast cancer outcomes in women visiting physiotherapist

Women visiting physiotherapist (*n* = 409) s		
Sociodemographic characteristics		
Age (years) at the time of the survey, *n* (%) 29–54 55–62 63–70 71–95	126 128 87 54	31.9% 32.4% 22.0% 13.7%
Highest level of education attained, *n* (%) None or primary Secondary Superiors	43 137 210	11.0% 35.1% 53.8%
National Classification of Occupations, *n* (%) Manual workers Intermediate Directors and managers	92 132 154	24.3% 34.9% 40.7%
Mutual or private insurance, *n* (%) No Yes	146 260	36.0% 64.0%
Received information regarding medical care and rehabilitation, *n* (%) None of low Fair or too much	248 128	66.0% 34.0%
Breast cancer outcomes		
Cancer stage, *n* (%) In situ Initial Local Metastasis	30 135 199 4	8.2% 36.7% 54.1% 1.1%
Years from the diagnosis, *n* (%) 0–3 4–6 7–9 10–26	102 131 95 81	24.9% 32.0% 23.2% 19.8%
Number of relapses, *n* (%) None One More than one	156 44 209	38.1% 10.8% 51.1%
General health status, *n* (%)		
Low Good or excellent	27 94	22.3% 77.7%

(51.1%) also attended more frequently to physiotherapy. Given special attention to the information received regarding medical care, 66.0% of this visiting physiotherapist declared receiving none or low information. (51.1%) also attended more frequently to physiotherapy. Given special attention to the information received regarding medical care, 66.0% of this visiting physiotherapist declared receiving none or low information.

When analyzing the relationship between sample socio-economic characteristics and BC stage with the access to the physiotherapy services, it was observed between-group significant differences in terms of age (*p* < 0.001), level of education (*p* < 0.001), having mutual insurance (*p* = 0.014), cancer stage (*p* = 0.004), number of relapses (*p* = 0.008), and general health status (*p* < 0.001) (Table 2).

**Table 2:** Comparison of sociodemographic and data of breast cancer outcomes

Outcome	Total sample	Non-visiting physiotherapist	Visiting physiotherapist	Between-groups comparison (p-values)
Socio-economic characteristics				
Age (years) at the time of the survey, n (%) 29–54 55–62 63–70 71–95		435(24.3%) 445(24.9%) 427(23.9%) 461(25.8%)	126 (31.9%) 128 (32.4%) 87(22.0%) 54(13.7%)	< 0.001*
Total	2181	1768	395	
Highest level of education attained, n (%) None or primary Secondary Superiors		395 (23.2%) 627 (36.9%) 677 (39.9%)	39 (10.1%) 137 (35.5%) 210 (54.4%)	< 0.001*
Total	2085	1699	386	
National Classification of Occupations, n (%) Manual workers Intermediate Directors and managers		449 (29.1%) 546(35.4%) 547(35.5%)	92 (24.3%) 132(34.9%) 154(40.7%)	0.091
Total	1920	1542	378	
Mutual or private insurance, n (%) No Yes		513 (29.7%) 1215 (70.3%)	146 (36.0%) 260 (64.0%)	0.014*
Total	2134	1728	406	
Received information regarding rehabilitation, n (%) None or low Fair or too much		987 (65.7%) 515(34.3%)	248(66.0%) 128(34.0%)	0.93
Total	1878	1502	376	
Breast cancer outcomes				
Cancer stage, n (%) In situ Initial Local Metastasis		147 (9.3%) 728 (45.9%) 690 (43.5%) 20 (1.3%)	30 (8.1%) 135(36.7%) 199(54.1%) 4(1.1%)	0.004*
Total	1953	1585	368	
Years from the diagnosis, n (%) 0–3 4–6 7–9 10–26		386 (21.1%) 560 (30.7%) 453 (24.8%) 427 (23.4%)	102 (24.9%) 131 (32.0%) 95 (23.2%) 81 (19.8%)	0.20
Total	2235	1826	409	
Number of relapses, n (%) None One More than one		828 (45.3%) 135 (7.4%) 863 (47.3%)	156 (38.1%) 44 (10.8%) 209 (51.1%)	0.008*
Total	2235	1826	409	
General health status, n (%) Low Goo	Low Goo			
Low Good or excellent		571 (32.2%) 1200 (67.8%)	197 (50.4%) 194 (49.6%)	< 0.001*
Total	2186	1771	391	

All data are presented as number of sample (*n*) and percentages (%). *Student *t* test. Significant differences

### Clinical symptomology characteristics

Table 3 shows the results in terms of clinical symptomatology experienced by the women in the study during the last week (acute) and the last 12 months (chronic), segmented according to whether they had visited a physiotherapist. Arm pain (*n* = 1129) and chest pain (*n* = 1078) were the most frequently acute symptoms, as well as low back pain (*n* = 686) and reduced arm strength (*n* = 560), those experienced the most in the last 12 months. Both in cases of acute and chronic symptomatology, only a low percentage of women, between 20 and 35%, visited the physiotherapist, revealing significant differences between groups in the majority of the symptoms. Lymphedema (34.1%) and decreased hand grip (29.5%) were the chronic symptoms that, if experienced, received the most visits to the Physiotherapist, as well as for general pain (26.0%) and discomfort in daily activities (25,4%) in terms of acute symptomatology.

**Table 3:** Comparison of clinical symptomatology for acute period or chronic period for women visiting and non-visiting physiotherapy from the total sample (*n* = 2235)

Total sample *n* = 2235	No visiting physiotherapist (*n* = 1826)	Visiting physiotherapist (*n* = 409)	Between-groups comparison (*p*-values)
Acute period, *n* (%)			
Pain Discomfort in daily activities Arm pain Difficulties to raise the arm Swelling of the arm or hand Chest pain Chest swelling	719 (74.0%) 675 (74.6%) 870 (77.1%) 412 (74.9%) 577 (78.7%) 870 (80.7%) 340 (79.1%)	253 (26.0%) 230 (25.4%) 259 (22.9%) 138 (25.1%) 156 (21.3%) 208 (19.3%) 90 (20.9%)	< 0.001*
Chronic period, n (%)			
Cervical pain Low back pain Tendinopathy Arm pain Lymphedema Reduced arm strength Reduced hand grip	357(72.7%) 526(76.7%) 210(69.5%) 357 (71.7%) 245(65.9%) 414(73.9%) 184 (70.5%)	134 (27.3%) 160 (23.3%) 92 (30.5%) 141 (28.3%) 127 (34.1%) 146 (26.1%) 77 (29.1%)	0.190 0.017 < .0.001* < 0.001* < 0.001* 0.011* 0.240 0.120

### Influence of socio-economic, breast cancer and clinical outcomes on the variable visiting physiotherapy

In general, women with breast cancer who have some types of pain are more likely to go to the physiotherapist. In this sense, the proportion of women who went to the physiotherapist was 1.58 [95%CI:1.29, 1.93] times higher in women who present with chronic tendinitis than in those who did not. This result is similar to that of women presenting with lymphedema, pain during the previous week or arm pain where the Prevalence Ratios were 1.78 [95%CI:1.45, 2.11], 1.55 [95%CI:1.26, 1.88] and 1.32 [95%CI:1.08, 1.64], respectively (Table 4). However, the main finding was that regardless of the pain that women suffering from breast cancer might suffer or their clinical variables, people with most advantaged socio-economic position were the ones who visited the physiotherapist the most. In this sense, in women with university studies, the proportion of women who visited the physiotherapist was 2.08 [95%CI: 1.52, 2.83] higher than those with primary education level or less. In addition, among women who had a private mutual insurance company, the proportion of visits to the physiotherapist was 1.22 [95%CI: 1.02, 1.47] higher than among those who did not.

**Table 4:** Proportion of women (%) with breast cancer who visit a physiotherapist (Yes) according to different independent variables and associated factors

			Non Adjusted		Adjusted	
		%	IRR	IC95	PRa	IC95
Acute period symptoms						
Pain	Yes	26.0	1.91	[1.59, 2.29]	1.55	[1.26, 1.88]
	No	13.7	1			
Discomfort in daily activities	Yes	25.4	1.75	[1.46, 2.10]		
	No	14.5	1			
Arm pain	Yes	22.9	1.69	[1.41, 2.03]	1.32	[1.08, 1.64]
	No	13.6	1			
Difficulties to raise the arm	Yes	25.1	1.56	[1.30, 1.87]		
	No	16.1	1			
Hand or arm swelling	Yes	21.3	1.26	[1.06, 1.51]		
	No	16.8	1			
Chest pain	Yes	19.3	1.11	[0.93, 1.32]		
	No	17.4	1			
Chest swelling	Yes	20.9	1.18	[0.96, 1.46]		
	No	17.7	1			
Chronic period symptoms						
Cervical pain	Yes	27.3	1.73	[1.44, 2.07]		
	No	15.8	1			
Low back pain	Yes	23.3	1.45	[1.21, 1.73]		
	No	16.1	1			
Tendinitis	Yes	30.5	1.86	[1.52, 2.26]	1.58	[1.29, 1.93]
	No	16.4	1			
Arm Pain	Yes	28.3	1.88	[1.54, 2.29]		
	No	15.0	1			
Lymphedema	Yes	34.1	2.26	[1.89, 2.69]	1.78	[1.45, 2.11]
	No	15.1	1			
Reduced arm strength	Yes	26.1	1.66	[1.39, 1.99]		
	No	15.7	1			
Reduced hand grip	Yes	29.5	1.75	[1.42, 2.17]		
	No	16.8	1			
Socio-economic outcomes						
Age (years) at the time of the survey	29–54 years	22.5	1			
	55–62 years	22.3	0.99	[0.80, 1.23]		
	63–70 years	16.9	0.75	[0.59, 0.96]		
	71–95 years	10.5	0.46	[0.35, 0.26]		
Highest level of education attained	None or primary	9.8	1		1	
	Secondary	17.9	1.83	[1.32, 2.52]	1.66	[1.20, 2.28]
	Superior	23.7	2.41	[1.77, 3.28]	2.08	[1.52, 2.83]
National Classification of Occupation	Manual workers	17.0	1			
	Intermediate occupations	19.5	1.14	[0.90, 1.46]		
	Directors and managers	22.0	1.29	[1.02, 1.63]		
Mutual or private insurance	Yes	22.2	1.26	[1.05, 1.50]	1.22	[1.02, 1.47]
	No	17.6	1			
Received information to medical care and rehabilitation	None or low	20.1	1			
	Fair or too much	19.9	0.99	[0.82, 1.20]		
Breast cancer outcomes						
Cancer stage	In situ	16.9				
	Initial phase tumor	15.6	0.92	[0.64, 1.32]		
	Local tumor	22.4	1.32	[0.93, 1.87]		
	Metastasis tumor	16.7	0.98	[0.38, 2.55]		
Years from the diagnosis	0–3	20.9	1			
	4–8	19.0	0.91	[0.72, 1.14]		
	7–9	17.3	0.83	[0.64, 1.07]		
	10–26	15.9	0.76	[0.59, 0.99]		
Number of relapses	None	15.9	1		1	
	One	24.6	1.55	[1.15, 2.08]	1.2	[0.88, 1.64]
	More than one	19.5	1.23	[1.02, 1.48]	1.21	[1.01, 1.46]
General health status	Low	23.6	1			
	Good or excellent	16.5	0.70	[0.58, 0.84]		

## Discussion

This study was aimed to provide a comprehensive perspective on the utilization and accessibility of physiotherapy services, as well as their clinical and socio-economic determinants, among women diagnosed with breast cancer within the hospital network of Barcelona. The results from a general health questionnaire involving 2,235 women showed that, despite experiencing various clinical symptoms and functional limitations, overall, only a small percentage of these women (18%) had visited a physiotherapist in the last 12 months. Furthermore, disparities in access to physiotherapist for women with breast cancer based on socio-economic factors became evident. This inequality may clearly impact on their recovery and underscores the influence of social and economic context on health status, as has been postulated in other similar studies [18]. In addition, 2 out of every 3 women reported receiving insufficient information about medical care in rehabilitation, which may hinder the access to physiotherapy. The results derived from the present study may offer insight into the role and current use of physiotherapy among women diagnosed with breast cancer within the Barcelona network health system.

A large body of evidence indicates physiotherapy interventions to be effective for pain release and functional enhancement in breast cancer women [9, 23]. Paradoxically, in the present study, overall, only 1 in 4 women experiencing functional limitations attended to physiotherapy. These data highlight limited access to physiotherapy in the rehabilitation of breast cancer within the Spanish health system, which restricts the well-exposed potential benefits only to a small percentage of those who utilize these services. Low attendance to physiotherapy services among that population has also been reported in other countries’ health systems. In the survey study by Rangel et al. [9], only less than half of the breast cancer survivors have visited physiotherapy, but the 100% of the visiting-women considered it was essential to enhance quality of life and reduce sports and functional limitations. Low rate of access to physiotherapy or rehabilitation services is also reported by other studies [11], not only among breast cancer women, but also in other oncologic conditions [24]. Cheville et al. [25] found similar results to ours, indicating that fewer than one-third of remediable physical impairments related to breast cancer receive appropriate rehabilitation services. These findings underscore the imperative to address the constraints and barriers experienced by both patients and healthcare services. One of the reasons proposed to explain this limited access to physiotherapy is the lack of knowledge among health professionals regarding the appropriate utilization of physiotherapy in functional rehabilitation [26]. Some studies have reported that only in a quarter of the cases, functional impairments are detected by oncologist and referral to rehabilitation services is made [27]. This situation may lead to patients receiving insufficient or inadequate information about how to manage functional limitations. Precisely, in the present study, 2 out 3 women reported receiving insufficient information about the medical care. In view of this elevated percentage, it remains necessary to improve the communication both between different health professional and with the patients. Patient expectations, attitudes, and beliefs are shown to be factors underlying the low-rate access to physiotherapy and rehabilitation services among oncology patients [12, 14]. Therefore, creating a more fluid communication and providing more information to patients about their functional prognosis of evolution would help patients to better understand the role and the benefits that physiotherapy may play in their functional recovery.

Treatment-related sequelae and breast cancer-induced fatigue may lead to women diagnosed with breast cancer continuing to experience pain and functional limitations years after their diagnosis. Diverse studies report shoulder and arm pain in around 30% of the breast cancer survivors [28]. Low back pain and cervical pain are also found to be one the most prevalent complaints, with a prevalence rate about 30%, while lymphedema is present in nearly 25% [28]. In the present study, these symptoms and functional limitations were also found to be present among women with breast cancer, with similar rates of prevalence to those reported previously. This reflects the fact that in breast cancer, beyond the curative treatment of the oncological process, a physical recovery process is necessary to reach a correct functional state.

From the other side, the proportion of women attending physiotherapy varied according to the presence of different clinical symptoms, but surprisingly, the symptoms that were proportionally most treated by physiotherapy were not the most prevalent among the women surveyed. Concretely, 372 women declared to experience lymphedema in the last 12 months, with 34.1% of them visiting physiotherapy. Low back pain, reduced strength, and pain in the arm were more prevalent in the studied cohort, although the proportion of visiting-physiotherapy women was of 23.3%, 26.1%, and 28.3%, respectively. The occurrence of lymphedema following surgical treatment of breast cancer is well known and potential benefits from physiotherapy treatment are well established. This is probably translated into a greater patient awareness of both the potential occurrence of lymphedema and effectiveness of physiotherapy intervention and contributes to an easier access to physiotherapy services. Other symptoms, such as low back pain or decreased arm strength, may be less likely to be attributed by the patient to the cancer process, as well as being less aware of how physical therapy can help treat them. Precisely, other studies point out that the perception of not needing treatment is one of the reasons why oncology patients do not go to the physiotherapist and could be related to the results obtained in this study [14].

The number of relapses may influence access to physiotherapy, which is consistent with other studies showing that a higher number of relapses require more medical attention [30]. However, the type of surgery and the amount of treatment required also appear to influence clinical status, as suggested by other studies [31], and may also impact on the need for physiotherapy, although this was not assessed in this study.

Socio-economic factors influenced the access to physiotherapy, being women with a higher level of education, occupation, and ability to purchase private insurance the most likely to visit a physiotherapist, being consistent with previous studies [18]. Graells-Sans et al. and Puigpinós-Riera et al. [31, 32]. have similarly employed these attributes to delineate the socio-economic profile of women with breast cancer, highlighting their relevance and usefulness in portraying the socio-economic status of the participants. Furthermore, additional studies such as Usera-Calvero et al. [33] and Sotas et al. [20] have incorporated factors such as social network, cohabitation, and partner or spouse income as additional features to determine socio-economic status. The findings of this study align with existing research, reinforcing the observation that women with lower social status and fewer economic incomes exhibit diminished access to healthcare services [33, 34] and display lower compliance with medical recommendations [20]. Indeed, low social status has been associated with greater difficulties for optimal recovery from breast cancer, experiencing higher levels of anxiety and depression [32] and lower quality of life [31]. Lower purchasing power, which limits the access to non-public physiotherapy services, coupled with reduced awareness of public health issues and biomedical information, as well as a greater logistical difficulties in accessing health services may account for these class-based differences [18].

While Spanish healthcare is universal and free of access to all citizens, the social inequalities on its utilization and access observed in the present study, together with previous evidence, challenge this paradigm [35]. With such a clear impact on potential recovery, special attention should be given to reducing social inequalities in healthcare system access and promoting policies to facilitate access to physiotherapy in all socio-economic profiles of women. The first step should be to recognize and identify this specific demographic profile in order to promote strategies that improve access for those with lower social levels [18], especially in the light of the increasing number of breast cancer diagnoses in most Western countries [1]

This study has implications for both research and clinical practice. Firstly, this study provides new evidence in the context of the Spanish healthcare system from a large cohort of women diagnosed with breast cancer and may serve as a basis for further research to detail clinical and socio-economic parameters. In terms of clinical application, access to physiotherapy should be promoted by doctors, oncologists, and other health professionals, and potential barriers should be identified and addressed.

The present study is limited by the fact that it is a cross-sectional study and, therefore, it was not possible to analyze how outcomes evolve over the breast cancer treatment period. In addition, the analysis has focused on the results obtained from the variables assessed, and there may be other factors that also influence access to a physiotherapist, which have not been considered in this study. Specifically, other socio-economic factors, such as cohabitation and caregiving responsibilities, as well as clinical and treatment-related outcomes, such as number of chemotherapy sessions received or the need for additional surgery, were not available for the current analysis but may play a role, as suggested by previous studies [31, 33] Future research should include these outcomes to delve deeper into the clinical and socio-economic influences on functional limitations and access to physiotherapy for women with breast cancer [26, 31]. Additionally, it is worth noting that the cohort from which the information for this study was partly collected data from patients in a self-reported manner. Among other considerations, the patient’s perception of pain and functionality may be influenced by psychological aspects.

## Conclusions

Although a significant proportion of women diagnosed with breast cancer exhibit functional limitations, only a small percentage of them have access to physiotherapy. Socio-economic factors such as education, occupation, and economic status along with insufficient information received and clinical factors hinder their access. Promoting strategies to reduce social biases as well as improve communication and patient information regarding physiotherapy may be of interest in enhancing healthcare for women diagnosed with breast cancer.

## Data Availability

The datasets generated during and/or analyzed during the current study are not publicly available due to ethics restrictions but are available from the corresponding author under reasonable request.
